# Integrative perspective of the healthy aging process considering the metabolome, cardiac autonomic modulation and cardiorespiratory fitness evaluated in age groups

**DOI:** 10.1038/s41598-022-25747-5

**Published:** 2022-12-09

**Authors:** Étore De Favari Signini, Alex Castro, Patrícia Rehder-Santos, Juliana Cristina Millan-Mattos, Juliana Magalhães de Oliveira, Vinicius Minatel, Camila Bianca Falasco Pantoni, Heloisa Sobreiro Selistre de Araújo, Fernando Fabrizzi, Alberto Porta, Antônio Gilberto Ferreira, Regina Vincenzi Oliveira, Aparecida Maria Catai

**Affiliations:** 1 Department of Physiotherapy, Federal University of São Carlos, São Carlos, São Paulo Brazil; 2 Department of Chemistry, Federal University of São Carlos, São Carlos, São Paulo Brazil; 3 Department of Gerontology, Federal University of São Carlos, São Carlos, São Paulo Brazil; 4 Department of Physiological Sciences, Federal University of São Carlos, São Carlos, São Paulo Brazil; 5 Penápolis Educational Foundation (FUNEPE), Penápolis, São Paulo Brazil; 6 Department of Biomedical Sciences for Health, University of Milan, Milan, Italy; 7 Department of Cardiothoracic, Vascular Anesthesia and Intensive Care, Policlinico San Donato, San Donato Milanese, Milan Italy; 8 Cardiovascular Physical Therapy Laboratory, Department of Physical Therapy, Nucleus of Research in Physical Exercise, Federal University of São Carlos, Via Washington Luiz, Km 235, CP: 676, São Carlos, SP 13565-905 Brazil

**Keywords:** Ageing, Metabolomics, Nonlinear dynamics

## Abstract

The aging process causes changes at all organic levels. Although metabolism, cardiac autonomic modulation (CAM), and cardiorespiratory fitness (CRF) are widely studied as a function of age, they are mainly studied in isolation, thus making it difficult to perceive their concomitant variations. This study aimed to investigate the integrated changes that occur in the metabolome, CAM, and CRF throughout aging in apparently healthy individuals. The subjects (n = 118) were divided into five groups according to age (20–29, 30–39, 40–49, 50–59, and 60–70 years old) and underwent blood collection, autonomic assessment, and a cardiopulmonary exercise test for metabolomics analysis using mass spectrometry and nuclear magnetic resonance, cardiac autonomic modulation analysis, and CRF by peak oxygen consumption analysis, respectively. The Tukey’s post hoc and effect size with confidence interval were used for variables with a significant one-way ANOVA effect (*P* < 0.01). The main changes were in the oldest age group, where the CRF, valine, leucine, isoleucine, 3-hydroxyisobutyrate, and CAM reduced and hippuric acid increased. The results suggest significant changes in the metabolome, CAM, and CRF after the age of sixty as a consequence of aging impairments, but with some changes in the metabolic profile that may be favorable to mitigate the aging deleterious effects.

## Introduction

Aging is a complex process characterized by changes at all organic levels^1^. There are several hallmarks of senescence that can be summarized in genomic instability, telomere attrition, epigenetic alterations, loss of proteostasis, unregulated nutrient sensing, mitochondrial dysfunction, cellular senescence, stem cell exhaustion, and altered intercellular communication^2,3^. The first four hallmarks are primarily responsible for the deleterious effects of aging, the next three are the positive or negative influencers of the four previous hallmarks, while the last two are consequences of changes in the previous seven hallmarks, and provide feedback on the deleterious effects^2,3^.

These alterations observed during aging are related to functional and structural changes in organic systems^3–7^ such as the integrity of the autonomic nervous system (ANS) and the body’s ability to generate energy using oxygen^7–10^. While the ANS plays a role in controlling, maintaining, and regulating vital and visceral functions in the body^11^, the peak oxygen consumption (VO_2PEAK_) is a result of the greatest capacity of the integrated activity of metabolism with the muscular, cardiovascular, respiratory, and nervous systems and is related with cardiorespiratory fitness (CRF)^12^. Thus, the activity of the ANS and the VO_2PEAK_ value are important markers of systemic and metabolic integrity and health. Several studies reported an imbalance in ANS, such as an increase in cardiac sympathetic modulation (CSM), a reduction in cardiac parasympathetic modulation (CPM), and a progressive reduction in VO_2PEAK_ values with physiological aging^8,9,13^.

Recently, with the advance of bioinformatics and “omics” sciences, several studies have used metabolomics to study metabolism during aging, especially due to its advantage of evaluating the phenotypic characteristics of the organism^1,14–16^. The main focus of these studies is on which alterations and profiles in the human metabolome are related to aging and longevity^14,16^. Thus, studies have also investigated the associations between organic system changes commonly observed during aging and the metabolic profile^14,17^, including the relationship between metabolome and cardiorespiratory fitness, or metabolome and ANS control^18–20^. Studies showed differences in serum levels of metabolites involved mainly in bioenergetic pathways in individuals with greater cardiorespiratory fitness (such as reductions in amino acids, intermediates of the citric acid cycle, and glycerol in blood at rest)^21,22^. On the other hand, considering the cardiac autonomic control in a disease context such as diabetes mellitus, studies showed a serum imbalance of amino acids, fatty acids, and metabolites involved in the citric acid cycle with a reduction in CPM^18,23^. However, little is known about the integrative changes in the metabolic profile and organic systems during healthy aging in individuals without major cardiovascular risk factors (e.g., obesity, hypertension, tabagism, etc.).

Considering the current emerging interest in understanding the complexity of the aging process, the joint assessment of the metabolic profile with cardiac autonomic modulation (CAM) and cardiorespiratory fitness over the decades of life can provide a broader understanding of the changes caused by physiological aging. Furthermore, this knowledge will allow us to verify if there is an age group where the alterations are more evident, which may contribute to the development of more effective strategies for healthy aging. In this context, the purpose of this study is to investigate the time course of variables in the human metabolome, as well as variables related to CAM and cardiorespiratory fitness, throughout the aging process in apparently healthy individuals without major risk factors.

## Methods

### Participants

**Figure 1:**
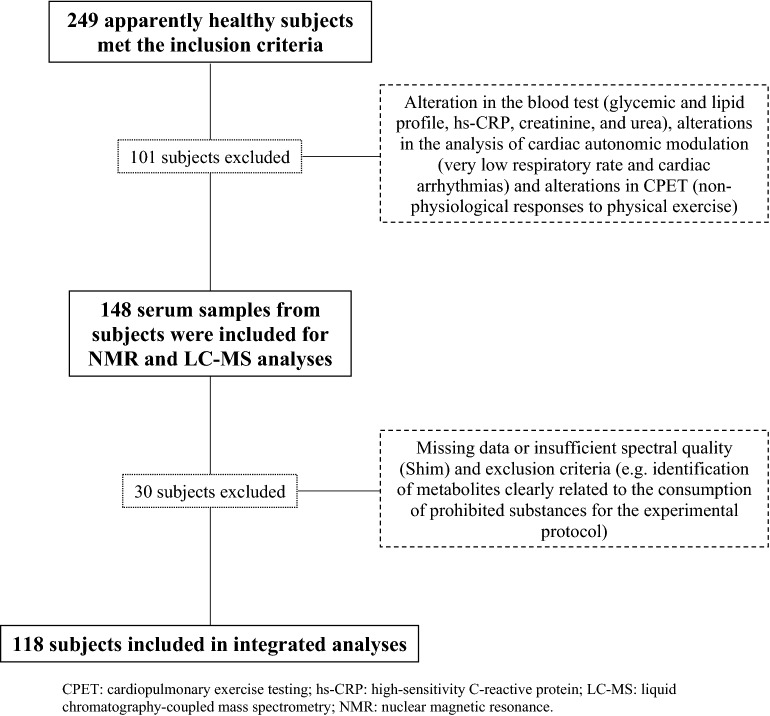
Loss flowchart. *CPET* cardiopulmonary exercise testing, *hs-CRP* high-sensitivity C-reactive protein, *LC–MS* liquid chromatography-coupled mass spectrometry, *NMR* nuclear magnetic resonance.

Participants were recruited through electronic and print-based media, as well as through contacts using the Cardiovascular Physical Therapy Laboratory (LFCV) database at the Federal University of São Carlos (UFSCar), São Carlos, Brazil. Anamnesis and a physical exam [acquisition of weight, height, and body mass index (BMI)] were performed and all subjects were screened using a generic questionnaire that included questions on: history of diseases and medical conditions, use of regular medications, previous clinical examinations, history of family diseases, performing specific diets, and physical activity. Subjects were included if they were apparently healthy (without any health conditions, such as cardiovascular, respiratory, musculoskeletal, metabolic, and neurological issues); non-obese (BMI < 30 kg/m^-2^); non-smokers; non-alcoholics or users of illicit drugs or regular medications related to chronic conditions; and free from any history of cardiovascular disease. The subjects included were asked to perform an ergometric test with a cardiologist at the UFSCar School Health Unit before starting the experimental protocol if they had not performed the exam recently (< 1 year). All subjects who had cardiovascular alterations such as excessive arrhythmias, myocardium ischemic signals (ST segment depression) or blood pressure hyperreactivity (excessive or non-exercise-proportional increase in blood pressure), verified by alterations in electrocardiogram (ECG) signals and in measuring blood pressure respectively, in the ergometric test or in the cardiopulmonary exercise test (CPET), as well as severe or recurrent hypotension, and evident blood test alterations during the experimental protocol (e.g., hyperglycemia and high level of C-reactive protein) were excluded. Then, one hundred and eighteen individuals, apparently healthy, aged from 20 to 70 years old participated in the study (Fig. 1, Table 1). They were divided into five groups according to age: 20–29 years old (G_20–29_), 30–39 years old (G_30–39_), 40–49 years old (G_40–49_), 50–59 years old (G_50–59_), and 60–70 years old (G_60–70_). The study was approved by the Human Research Ethics Committee at UFSCar (number: 173/2011) and conducted in accordance with the standards set by the Declaration of Helsinki. All participants signed a free and informed consent form after accepting to participate in this study.

**Table 1:** Characteristics of the participants.

Characteristics	All	G_20–29_	G_30–39_	G_40–49_	G_50–59_	G_60–70_
Participants (n)	118	29	27	30	14	18
Age (years)	41 ± 14	25 ± 2	33 ± 3	44 ± 3	53 ± 3	65 ± 3
Men/women (ratio)	1.31	0.93	1.45	2.33	1.80	0.64
Height (m)	1.68 ± 0.09	1.69 ± 0.08	1.70 ± 0.09	1.68 ± 0.07	1.69 ± 0.08	1.63 ± 0.08
Weight (kg)	70.66 ± 12.56	67.93 ± 13.20	71.70 ± 13.18	73.48 ± 12.44	70.30 ± 11.30	69.10 ± 11.81
BMI (kg/m^2^)	24.65 ± 2.92	23.36 ± 2.84	24.39 ± 2.67	25.63 ± 3.08	24.41 ± 2.73	25.68 ± 2.59

Data are mean ± standard deviation.

*BMI* body mass index, *G*_*20–29*_ 20–29 years old group, *G*_*30–39*_ 30–39 years old group, *G*_*40–49*_ 40–49 years old group, *G*_*50–59*_ 50–59 years old group, *G*_*60–70*_ 60–70 years old group.

### Experimental design

Anamnesis and the physical exam were performed at least 2 days before the experimental protocol. Except for blood collection, all tests were performed at LFCV. Blood collection for metabolomic and biochemical analysis was performed in a specialized laboratory in São Carlos (UNIMED Clinical Analysis Laboratory of São Carlos).

### Blood samples

On the same day as the autonomic assessment, the blood collection was performed after 12-h of fasting in the morning. The blood samples were used for metabolomic analysis and for performing biochemical tests in the specialized laboratory to assess the participants’ health status. For metabolomics, blood samples collected in serum separator tubes (S-Monovette 4.9 mL, Sarstedt, Germany) were immediately taken to the Physiotherapy Department. The blood samples were then centrifuged at 1450×*g* for 10 min (Sorvall ST 8 Benchtop Centrifuge, Thermo Scientific, Massachusetts, USA), and the serum was collected and stored at − 80 °C for further analysis.

The health status of the participants was verified by the fasting values of total cholesterol (TC), very low density lipoprotein (VLDL), low density lipoprotein (LDL), high density lipoprotein (HDL), triglycerides, glucose, uric acid, urea, creatinine, and high-sensitivity C-reactive protein (hs-CRP) (Supplementary Table S1). The TC, HDL, VLDL, triglycerides, uric acid, urea, glucose, and creatinine were measured using wet chemistry (except for LDL which was calculated from the Friedewald equation) (Advia 1800, Siemens, Germany). The hs-CRP was quantified by turbidimetry (Advia 1800, Siemens, Germany).

### Cardiac autonomic assessment

The autonomic cardiac assessment was always carried out in the afternoon. Room temperature was maintained between 21 and 24 °C and relative humidity was between 40 and 60%. All participants were instructed to avoid the consumption of stimulants, heavy meals, and alcoholic drinks for at least 24 h before the test and avoid strenuous physical activity for at least 48 h before the test^24^. In addition, women of reproductive age were evaluated only in the follicular period of the menstrual cycle (ranging from the 7th and 10th day of the menstrual cycle)^25^. Only women of reproductive age with regular menstrual cycles or postmenopausal women (characterized by amenorrhea by at least 1 year) were included. However, women with the criteria described above and who were using contraceptives or taking hormone replacement therapy were not included in the study^25^. The test included the acquisition of cardiovascular control markers at rest supine position. The participant was maintained at rest in the supine position on a stretcher for 10 min to stabilize the cardiovascular variables. Then, cardiovascular and respiratory variables (heart rate, blood pressure, and respiratory rate) were continuously recorded for 15 min. All subjects were instructed to avoid any communication and movements during the whole test, except in necessary situations (e.g., physical discomfort). The ECG signals were acquired by MC5 lead (BioAmp FE132, ADInstruments, New South Wales, Australia); non-invasive continuous finger arterial pressure (Finometer Pro, Finapres Medical Systems, Netherlands), and respiratory movement through a thoracic belt (Marazza, Monza, Italy). All signals were integrated by hardware (Power Laboratory 8/35, ADInstruments, New South Wales, Australia) and processed by software LabChart, version 7.3.8 (ADInstruments, New South Wales, Australia).

### Cardiopulmonary exercise testing (CPET)

The CPET was performed on the same day as the blood test, but after the autonomic assessment, or on a close day to assess the cardiorespiratory fitness of the participants, defined as the peak oxygen consumption (VO_2PEAK_). All instructions and the room temperature and humidity control, which were used for cardiac autonomic assessment, were the same for the CPET. The CPET was carried out on a treadmill ergometer (Master ATL, Inbramed, Rio Grande do Sul, Brazil) with an incremental protocol^26^ until voluntary exhaustion or with the presence of interruption criteria proposed by Balady et al.^27^. Ventilatory and metabolic variables were obtained, breath-by-breath, through a metabolic cart (ULTIMA MedGraphics—St Paul, Minesota, USA) and processed using specific software (Breeze Suite 7.1, MedGraphics—St. Paul, Minesota, USA), while the 12-lead ECG was obtained by an electrocardiograph (CardioPerfect, Welch Allyn, New York, USA). The highest value of VO_2_ obtained in the last 30 s of the CPET was considered the VO_2PEAK_^26^.

### Autonomic data processing

The CAM analysis was performed on short-term sequences [256 consecutive heart period (HP) values] selected from the most stable part of the tachogram^28^. HP was obtained by the temporal distance between two consecutive normal R-wave peaks acquired in the ECG (i.e., the temporal distance between two consecutive heart beats in milliseconds), and the tachogram is the graphic reproduction of all HP values generated from each heartbeat over all analyzed heart beats. The tachogram of all participants was carefully checked to avoid erroneous detections or missed beats due to alterations in the delineations of the R-wave before selecting the sequences. The isolated ectopic beats that affected HP were corrected by linear interpolation using the most adjacent HP value unaffected^24,26^. Time domain variables, such as the HP mean and the HP variance, were calculated for each short-term sequence. The HP variance indicates global CAM (CPM + CSM).

Spectral analysis was performed by adjusting the univariate parametric spectral power of the short-term sequences according to the autoregressive model^29^. Then, the spectral components were decomposed into bands expressed in absolute units (ms^2^) and defined as high frequency bands (> 0.15 to 0.40 Hz), low frequency bands (0.04 to 0.15 Hz), and very low frequency (< 0.04 Hz)^28^. The analysis of CAM was based on the high (HF, which indicates CPM) and low (LF, which indicates the contribution of CSM plus CPM, with CSM predominance) absolute frequency bands. The ratio between these two indices (LF/HF) to typify the sympatho-vagal balance over the heart^30^ and the normalized index of each band (relative value in percentage of HF and LF in proportion to the total spectral power minus the very low frequency band) were also calculated.

### Metabolomics analyses

The metabolomics analysis was performed using liquid chromatography-high resolution (LC-HRMS) and proton nuclear magnetic resonance (^1^H NMR) with an untargeted approach.

#### ^1^H NMR

Considering ^1^H NMR, initially all serum samples were filtered in 3 kDa filters (Amicon Ultra) in centrifugation at 14,000×*g* for 30 min at 4 °C. The filters were previously washed five times with 500 μL of Milli-Q water, followed by centrifugation at 14,000×*g* for 5 min at 4 °C, and spinning (filter reverse and rotation at 7500×*g* for 60 s) to eliminate any residue of Milli-Q water. The filtered samples were transferred to 5-mm NMR tubes (Wilmad Standard Series 5 mm, Sigma-Aldrich) containing phosphate buffer [(monobasic sodium phosphate, NaH_2_PO_4_, 119.97 g/mol; dibasic sodium phosphate, Na_2_HPO_4_, 141.96 g/mol), TMSP-d4 (3-(trimethylsilyl)-2,2′,3,3′-tetradeuteropropionic acid) at 5 mmol/L as an internal reference], and D_2_O (99.9%; Cambridge Isotope Laboratories Inc.), with the respective proportions: 100 μL, 40 μL, and 260 μL. All the NMR measurements were acquired from a 14.1 Tesla Bruker spectrometer (600 MHz for hydrogen frequency), equipped with a 5 mm TCI cryoprobe using temperature 298 K. For the ^1^H spectrum a pulse sequence with H_2_O presaturation signal (named by Bruker as noesypr1d) was used adopting a continuous wave, assuming the following acquisition parameters: acquisition time (AQ = 3.63 s), spectral width (SW = 30 ppm), relaxation delay (d1 = 4 s), the 90° pulse time (p1 = 9.5 μs) and a number of scans (ns = 128). All spectra were processed with 0.3 Hz line broadening (lb) to attenuate the noise in the spectral signals. After spectrum acquisition, baseline corrections, identification, and quantification of metabolites present in the samples were conducted using Suite 8.6 Chenomx software (Chenomx Inc., Edmonton, AB, Canada) by the TMSP-d4 (0.5 mmol/L) signal as an internal reference to quantify other metabolites. Moreover, 2D NMR spectra (COSY, HSQC and HMBC) were used to confirm the identification made by Chenomx or the identification of other compounds.

#### LC-HRMS

Serum samples, stored at − 80 °C, were firstly thawed on ice and vortexed for 15 s. Afterward, the samples were submitted to the protein precipitation process. An aliquot of 150 μL of the sample was transferred to the new centrifuge tubes, and 450 μL of cold methanol was added to the tubes to initiate the protein precipitation and metabolite extraction. The mixture was stored at − 20 °C for 5 min. The centrifuge tubes were vortexed for 20 s and centrifuged at 7267×*g* at 4 °C for 10 min. Next, aliquots of 200 µL of the supernatant were transferred to the new centrifuge tubes and 20 µL of the internal standard (5 mmol/L of anhydrous l-Leucine-enkephalin acetate) were added to the samples and stored at − 20 °C until further analysis by LC-HRMS. A blank sample was prepared with 100 μL of methanol. Quality control (QC) samples were prepared from aliquots of 15 μL of the all the serum samples that had already been subjected to the protein precipitation process as described above and were injected in triplicate throughout the batch of experimental samples.

The UHPLC Agilent system (model 1290 Infinity II, Agilent) consisted of a binary LC-G712A pump with a blend assist G7104A, a vial sampler LC injector G7129C, and a column compartment G7129B. HyStar workstation software was used for data acquisition (HyStar Version 3.2, Bruker Daltonics) and a Compass Data Analysis was used for data analysis and processing (DataAnalysis Version 3.2, Bruker Daltonics). Chromatographic analyses were performed with an Eclipse SDB-C18 Agilent column (100 × 3.0 mm i.d; 3.5 μm) employing a gradient elution using water + 0.1% formic acid (solvent A) and acetonitrile + 0.1% formic acid (solvent B) as the mobile phase at a flow rate of 0.4 mL/min and temperature set at 40 °C. The total run time was 30 min using the following multistep gradient: 0 min, 1% B; 0–3.0 min, 1–2% B; 3–10 min, 2–30% B; 10–15 min, 30–50% B; 15–18 min, 50–80% B; 18–20 min, 80–90% B; 20–22 min, 90–95% B; 22–26 min, 95–99% B; 26.01–28 min, 99% B, for column cleaning and a conditioning cycle time of 3 min with the same initial conditions of 1% B. The injection volume was 5 μL.

The separated compounds were monitored with a quadrupole time-of-flight mass spectrometer (QqTOF-MS). The MS and MS/MS analyses were performed using an Impact HD QTOF™ mass spectrometer (Bruker Daltonics, Bremen, Germany) equipped with an ESI interface operating in negative or positive ion mode. The MS and MS/MS data were acquired through Compass QtofControl v.3.4 (Bruker Daltonik, Bremen, Germany) and the data were processed using Data Analysis 4.2 software (Bruker Daltonik). The ion source optimal parameters were set as follows: capillary voltage, + 3600 V and − 3000 V for the positive and negative ion modes, respectively. All other parameters were the same for both ion modes used: end plate offset, 450 V; nebulizer, 4 bar; dry heater temperature, 180 °C; dry gas flow, 8 L/min; and full-MS scan range, m/z 50–1300.

A data dependent acquisition (DDA) was used for the MS/MS analysis where the collision RF was set to vary from 200.0 to 550.0% Vpp; the transfer time was set to vary from 50.0 to 90.0 µs; with 50.0% timing each. The funnel RF 1 and 2 were 250.0 and 150.0 Vpp, respectively. The hexapole RF was 50.0 Vpp and the quadrupole ion energy was 5.0 eV with pre-pulse storage of 6.0 µs. Quadrupole ion energy and collision cell energy were both set at 5 eV. The parameters used to trigger the MS/MS fragmentation were 2.0 Hz for low counts (10,000 counts/per 1000 sum) and 4.0 Hz for high counts (100,000 counts/per 1000 sum), using a total cycle time of 3 s; absolute threshold of 1491 counts (302 counts/per 1000 sum), active exclusion 1 spectrum; release after 0.90 min, while the full MS acquisition was set at 2.0 Hz. The collision energy used for ion fragmentation was programmed to vary from 250.0 to 100.0% of the 20 eV initially set, with the following isolation mass: *m/z* 100, 200, and 300: 4 width; for *m/z* 700 and 1000: 6 width. Internal mass-spectrometer calibration was performed with 1 mmol/L of sodium formate prepared in acetonitrile, using a quadratic high-precision calibration (HPC) regression model. The calibration solution was injected at the end of each analytical run and all spectra were recalibrated before compound identification.

Bruker Profile Analysis v2.1 software was used to process the UHPLC-HRMS data. The bucket generation was performed under the following parameters: S/N threshold = 2; correlation coefficient threshold = 0.2; minimum compound length = 10 spectra; smoothing width = 1. All features detected by the LC-HRMS were subjected to data processing consisting of the inclusion of features based on: values greater than 5% of the values from blank samples; coefficient of variation (CV) of QCs samples (mean of replicates) lower than 20%; missing data lower than 10% in experimental samples. The remaining features were normalized by non-linear local regression (Loess) to verify the instrumental stability using the 1.1 software^31,32^ (Supplementary Fig. S1) and further analysis. Data Analysis v4.2 (Bruker Daltonik) was used to perform the identification of MS/MS fragments of identified peaks that were putatively confirmed by comparing fragments in the HMDB MS/MS database (https://hmdb.ca), Mass Bank (https://massbank.eu/MassBank/), CEU Mass Mediator (http://ceumass.eps.uspceu.es/) databases and based on the adducts: [M + H]^+^, [M + H − 2H_2_O]^+^, [M + H − H_2_O]^+^, [M + NH_4_ − H_2_O]^+^, [M + NH4]^+^, [M + Na]^+^, [M + CH_3_OH + H]^+^, [M + K]^+^, [M + ACN + H]^+^, [M + 2Na − H]^+^, [M + IsoProp + H]^+^, [M + ACN + Na]^+^, [M + 2K − H]^+^, [M + 2ACN + H]^+^, [M + IsoProp + Na + H]^+^, [M + H + HCOONa]^+^, [2M + H]^+^, [2M + NH_4_]^+^, [2M + Na]^+^, [2M + 2H + 3H_2_O]^+^, [2M + K]^+^, [2M + ACN + H]^+^, [2M + ACN + Na]^+^, [2M + H − H_2_O]^+^, [M + 2H]^+^, [M + H + NH_4_]^+^, [M + H + Na]^+^, [M + H + K]^+^, [M + ACN + 2H]^+^, [M + 2Na]^+^, [M + H + Na]^+^, [M + 2ACN + 2H]^+^, [M + 3ACN + 2H]^+^, [M + 3H]^+^, [M + 2H + Na]^+^, [M + H + 2Na]^+^, [M + 3Na]^+^, and [M + H + 2K]^+^ for the positive mode; and [M − H]^−^, [M − H_2_O − H]^−^, [M − Na − 2H]^−^, [M + Cl]^−^, [M + K − 2H]^−^, [M − FA − H]^−^, [M − Hac − H]^−^, [M − TFA − H]^−^, [M − H + HCOONa]^−^, [2M − H]^−^, [2M + FA − H]^−^, [2M + Hac − H]^−^, [3M − H]^−^, and [M − 3H]^−^ for the negative mode.

### Statistical analyses

Prior to the data analysis, Shapiro–Wilk and Levene tests were used to check the normality of data distribution and variance homogeneity assumptions, respectively. When variables did not reach these assumptions, data transformation including natural logarithmic (LN_X_), inverse (1/x), root cubic ($\sqrt[3]{x}$), root square $(\sqrt[2]{x})$, or quadratic (x^2^) transformations were applied, but all data were presented in their original scale for an easier interpretation^33^. The Chi-square test was used to compare categorical variables between groups. To compare the continuous variables between groups, the one-way ANOVA test, followed by the Tukey’s post-hoc test, was used when the normality of data distribution and variance homogeneity assumptions were confirmed, or the Kruskal–Wallis test followed by the Mann–Whitney test when the assumptions were violated despite data transformation. Assuming that a large number of statistical tests were performed, the significance level threshold was adjusted at a nominal value of *P* < 0.01 for a two-tailed test, recognizing that a full Bonferroni adjustment would probably reduce the discovery of false-negative observations, as it is too conservative. To supplement this approach, the effect size (ES: mean difference between groups divided by pooled standard deviation from all subjects) with a 99% confidence interval (99% CI) for each variable that presented a significant ANOVA effect was performed. When the 99% confidence interval of the effect size did not cross zero, the differences were also considered significant^34^. Afterward, a principal component analysis (PCA) was performed to characterize the groups based on significant variables. All the analyses described above were performed using *SPSS 25.0* software (Chicago, Illinois, USA).

## Results

### Sex and BMI influences

The chi-square test showed no significant sex influences (*P* = 0.220) in the age groups. Thus, statistically, there is a homogeneous distribution of men and women in each age group, allowing the exclusion of the influence of sex on the results. Considering BMI, there were no significant differences between age groups in the one-way ANOVA test (*P* = 0.019) allowing the exclusion of the influence of BMI on the results.

### Biochemical tests

All groups presented within the normal range or borderline values of biochemical variables^35–39^ (Supplementary Table S1). The significant ANOVA effects were only in hs-CRP (*P* = 0.003), TC (*P* < 0.001), LDL (*P* = 0.003), and urea (*P* = 0.006). For hs-CRP, the values were high in G_30–39_ (*P* = 0.009, ES_*d*_ = 0.922, CI99% = 0.184 to 1.659), G_50–59_ (*P* = 0.009, ES_*d*_ = 1.115, CI99% = 0.250 to 1.980), and G_60–70_ (*P* = 0.058, ES_*d*_ = 0.892, CI99% = 0.086 to 1.698) when compared to G_20–29_. The TC had higher values in older groups when compared to G_20–29_, as seen in G_40–49_ (*P* = 0.003, ES_*d*_ = 0.801, CI99% = 0.092 to 1.511), G_50–59_ (*P* = 0.016, ES_*d*_ = 0.909, CI99% = 0.063 to 1.754), and G_60–70_ (*P* = 0.003, ES_*d*_ = 0.956, CI99% = 0.145 to 1.767). For LDL, significantly high values were seen only in G_40–49_ (*P* = 0.006, ES_*d*_ = 0.877, CI99% = 0.162 to 1.591) and G_60–70_ (*P* = 0.018, ES_*d*_ = 0.930, CI99% = 0.121 to 1.739) when compared to G_20–29_. Finally, the urea was significantly higher in G_30–39_ (*P* = 0.048, ES_*d*_ = 0.856, CI99% = 0.123 to 1.589), G_50–59_ (*P* = 0.008, ES_*d*_ = 1.131, CI99% = 0.264 to 1.998), and G_60–70_ (*P* = 0.068, ES_*d*_ = 0.803, CI99% = 0.004 to 1.602) when compared to G_20–29_. All these results are presented in Table 2.

**Table 2:** Significant autonomic, metabolomic, and cardiorespiratory fitness variables across age groups.

Variables	Groups				
	G_20–29_	G_30–39_	G_40–49_	G_50–59_	G_60–69_
3-Hydroxyisobutyrate (mM)	0.014 (0.011–0.018)	0.016 (0.014–0.022)	0.015 (0.012–0.020)	0.015 (0.013–0.017)	0.012 (0.009–0.016)^bc^
Aspartate (mM)	0.024 (0.018–0.031)	0.025 (0.020–0.032)	0.032 (0.028–0.035)^ab^	0.031 (0.029–0.034)	0.031 (0.027–0.038)
Isoleucine (mM)	0.069 (0.060–0.082)	0.077 (0.065–0.084)	0.070 (0.061–0.086)	0.067 (0.059–0.081)	0.056 (0.049–0.063)^abcd^
Leucine (mM)	0.098 (0.075–0.112)	0.097 (0.087–0.116)	0.102 (0.084–0.120)	0.096 (0.082–0.112)	0.080 (0.070–0.092)^bc^
Valine (mM)	0.232 (0.204–0.274)	0.268 (0.237–0.298)^a^	0.249 (0.211–0.292)	0.246 (0.230–0.278)	0.198 (0.189–0.220)^abcd^
Hippuric acid (a.u.)	0.486 (0.389–0.692)	0.661 (0.433–0.977)	0.571 (0.394–0.855)	0.676 (0.538–0.771)	1.365 (0.864–2.502)^abcd^
10E,12Z-CLA (a.u.)	0.193 (0.129–0.287)	0.615 (0.195–1.587)^a^	0.831 (0.252–1.545)^a^	0.233 (0.132–0.471)	0.283 (0.139–0.383)^c^
VO_2PEAK_ (mL/kg/min)	32.300 (30.000–37.500)	37.000 (31.550–40.050)	30.950 (27.675–37.950)	29.500 (26.475–33.450)	23.850 (19.975–27.225)^abc^
hs-CRP (mg/L)	0.210 (0.070–0.500)	0.760 (0.235–1.505)^a^	0.370 (0.160–0.965)	1.190 (0.498–1.808)^a^	0.655 (0.228–1.128)^a^
TC (mg/dL)	171.000 (139.000–189.000)	179.000 (159.000–197.500)	191.500 (177.000–213.250)^a^	203.000 (170.250–207.000)^a^	194.000 (171.000–213.000)^a^
LDL (mg/dL)	93.000 (65.000–113.000)	102.000 (91.000–121.500)	118.500 (94.000–130.250)^a^	115.000 (110.000–132.500)	110.000 (100.500–131.500)^a^
Urea (mg/dL)	27.000 (23.000–32.000)	33.000 (29.500–37.000)^a^	32.000 (29.000–36.500)	34.000 (30.750–37.750)^a^	32.500 (29.250–36.500)^a^
HP variance (ms^2^)	2210.294 (1560.851–3355.204)	1709.864 (929.651–3496.373)	1345.621 (915.377–2210.501)	1189.985 (844.328–1843.296)^a^	825.298 (557.824–1243.271)^a^
LF (ms^2^)	811.257 (476.825–1302.738)	677.183 (264.036–1075.832)	460.334 (271.116–983.842)	373.885 (204.137–445.242)^a^	176.443 (72.286–385.287)^abc^
HF (ms^2^)	798.676 (429.766–1854.859)	517.024 (326.885–970.092)	340.342 (188.324–589.965)	364.898 (122.537–627.217)	176.384 (93.676–359.366)^ab^

Data presented in median and interquartile interval. Logarithm (for hippuric acid, 10E,12Z-CLA, isoleucine, 3-Hydroxyisobutyrate, leucine, HP Variance, LF, and HF), cubic root (for hs-CRP), quadratic (for TC), and inverse (for valine) transformations were used in the statistics. The variables not listed above were used the original data.

*10E,12Z-CLA* 10E,12Z-octadecadienoic acid, *G*_*20–29*_ 20–29 years old group, *G*_*30–39*_ 30–39 years old group, *G*_*40–49*_ 40–49 years old group, *G*_*50–59*_ 50–59 years old group, *G*_*60–70*_ 60–70 years old group, *HF* high frequency band, *HP* heart period, *hs-CRP* high-sensitivity C-reactive protein, *LDL* low density lipoprotein, *LF* low frequency band, *TC* total cholesterol, *VO*_*2PEAK*_ peak oxygen consumption.

^a^Difference with the G_20–29_ group.

^b^Difference with the G_30–39_ group.

^c^Difference with the G_40–49_ group.

^d^Difference with the G_50–59_ group.

Tukey’s post hoc at *P* < 0.01 and effect size with 99% CI for all variables with *P* < 0.01 in one-way ANOVA.

### CAM and CRF

Considering autonomic variables, only the LF, HF, and HP variance were significant (Table 2, Supplementary Table S2) among the age groups, for ANOVA effects (*P* < 0.001, *P* = 0.001, and *P* = 0.002 respectively). The oldest group had lower values of LF, HF, and HP variance when compared to the younger groups, considering the differences between G_60–70_ with G_20–29_ (*P* < 0.001, ES_*d*_ = − 1.349, CI99% = − 2.200 to − 0.498), G_30–39_ (*P* = 0.001, ES_*d*_ = − 1.076, CI99% = − 1.917 to − 0.235), and G_40–49_ (*P* = 0.007, ES_*d*_ = − 0.884, CI99% = − 1.680 to − 0.088) for LF, G_60–70_ with G_20–29_ (*P* = 0.004, ES_*d*_ = − 1.015, CI99% = − 1.831 to − 0.199) and G_30–39_ (*P* = 0.029, ES_*d*_ = − 0.946, CI99% = − 1.775 to − 0.117) for HF, and G_60–70_ with G_20–29_ (*P* = 0.001, ES_*d*_ = − 1.200, CI99% = − 2.035 to − 0.365) for the HP variance. For the LF and HP variance variables, lower values were also observed in the G_50–59_ with G_20–29_ (*P* = 0.085, ES_*d*_ = − 1.010, CI99% = − 1.865 to − 0.155 for LF, and *P* = 0.113, ES_*d*_ = -0.847, CI99% = − 1.687 to − 0.007 for the HP variance).

For CRF, the VO_2PEAK_ reduce with increasing age (ANOVA effect: *P* < 0.001) and indicating low values in the oldest group (Table 2) when compared to G_20–29_ (*P* = 0.001, ES_*d*_ = − 1.484, CI99% = − 2.352 to − 0.617), G_30–39_ (*P* < 0.001, ES_*d*_ = − 1.706, CI99% = − 2.624 to − 0.788), and G_40–49_ (*P* = 0.001, ES_*d*_ = − 1.233, CI99% = − 2.062 to − 0.404).

### Metabolomics

Considering the metabolomic results, the NMR technique identified 47 metabolites from the serum samples. However, only 5 metabolites were significant across the age groups (Table 2, Supplementary Tables S3, S4, Supplementary Fig. S2). Isoleucine (ANOVA effect: *P* < 0.001) had the lowest value in the G_60–70_ , with significant differences in G_20–29_ (*P* = 0.002, ES_*d*_ = − 1.197, CI99% = − 2.032 to 0.363), G_30–39_ (*P* < 0.001, ES_*d*_ = − 1.545, CI99% = − 2.441 to − 0.649), G_40–49_ (*P* < 0.001, ES_*d*_ = − 1.200, CI99% = − 2.026 to − 0.375), and G_50–59_ (*P* = 0.017, ES_*d*_ = − 1.010, CI99% = − 2.010 to − 0.010). Similarly, valine (ANOVA effect: *P* < 0.001) showed the lowest values in G_60–70_ , with higher values in G_20–29_ (*P* = 0.040, ES_*d*_ = − 0.826, CI99% = − 1.626 to − 0.025), G_30–39_ (*P* < 0.001, ES_*d*_ = − 1.747, CI99% = − 2.671 to − 0.823), G_40–49_ (*P* = 0.001, ES_*d*_ = − 1.101, CI99% = − 1.916 to − 0.285), and G_50–59_ (*P* = 0.006, ES_*d*_ = − 1.223, CI99% = − 2.249 to − 0.197), but also showed high values in G_30–39_ (*P* = 0.080, ES_*d*_ = 0.738, CI99% = 0.013 to 1.463) when compared to G_20–29_. Leucine (ANOVA effect: *P* = 0.003) had higher values in G_30–39_ (*P* = 0.007, ES_*d*_ = 1.049, CI99% = 0.211 to 1.888) and G_40–49_ (*P* = 0.002, ES_*d*_ = 1.057, CI99% = 0.245 to 1.868) compared to G_60–70_ . In addition, 3-hydroxyisobutyrate (ANOVA effect: *P* = 0.003), a valine derivative, had a similar pattern of leucine, with G_30–39_ (*P* = 0.001, ES_*d*_ = 1.219, CI99% = 0.363 to 2.074) and G_40–49_ (*P* = 0.031, ES_*d*_ = 0.844, CI99% = 0.051 to 1.637) with higher values than in the G_60–70_ . Finally, the non-essential amino acid aspartate (ANOVA effect: *P* = 0.007) was lower in G_20–29_ (*P* = 0.017, ES_*d*_ = − 0.827, CI99% = − 1.538 to − 0.116) and G_30–39_ (*P* = 0.067, ES_*d*_ = − 0.746, CI99% = − 1.464 to − 0.027) when compared to the G_40–49_ , and was slightly reduced in the subsequent age groups (ES_*d*_ = − 0.139 for G_50–59_ and ES_*d*_ = − 0.138 for G_60–70_ compared with G_40–49_) as a consequence of the loss of significance of the difference with the younger groups.

After applying the parameters for the inclusion of features, the LC-HRMS analysis technique identified 125 features. However, only 6 features were significant among the age groups, and only two were known metabolites (Table 2, Supplementary Tables S5, S6). The hippuric acid (ANOVA effect: *P* < 0.001) was higher in the G_60–70_ compared to the other groups: G_20–29_ (*P* < 0.001, ES_*d*_ = 1.389, CI99% = 0.533 to 2.245), G_30–39_ (*P* = 0.004, ES_*d*_ = 0.991, CI99% = 0.158 to 1.824), G_40–49_ (*P* = 0.001, ES_*d*_ = 1.170, CI99% = 0.347 to 1.992), and G_50–59_ (*P* = 0.009, ES_*d*_ = 1.056, CI99% = 0.051 to 2.061). The 10E,12Z-octadecadienoic acid (10E,12Z-CLA) (ANOVA effect: *P* = 0.001), a conjugated linoleic acid, was the other identified metabolite and showed higher values in the G_30–39_ (compared to G_20–29_: *P* = 0.019, ES_*d*_ = 0.816, CI99% = 0.086 to 1.546) and G_40–49_ (compared to G_20–29_: *P* = 0.004, ES_*d*_ = 0.956, CI99% = 0.236 to 1.677; G_60–70_: *P* = 0.054, ES_*d*_ = 0.815, CI99% = 0.024 to 1.606).

### Principal component analysis

**Figure 2:**
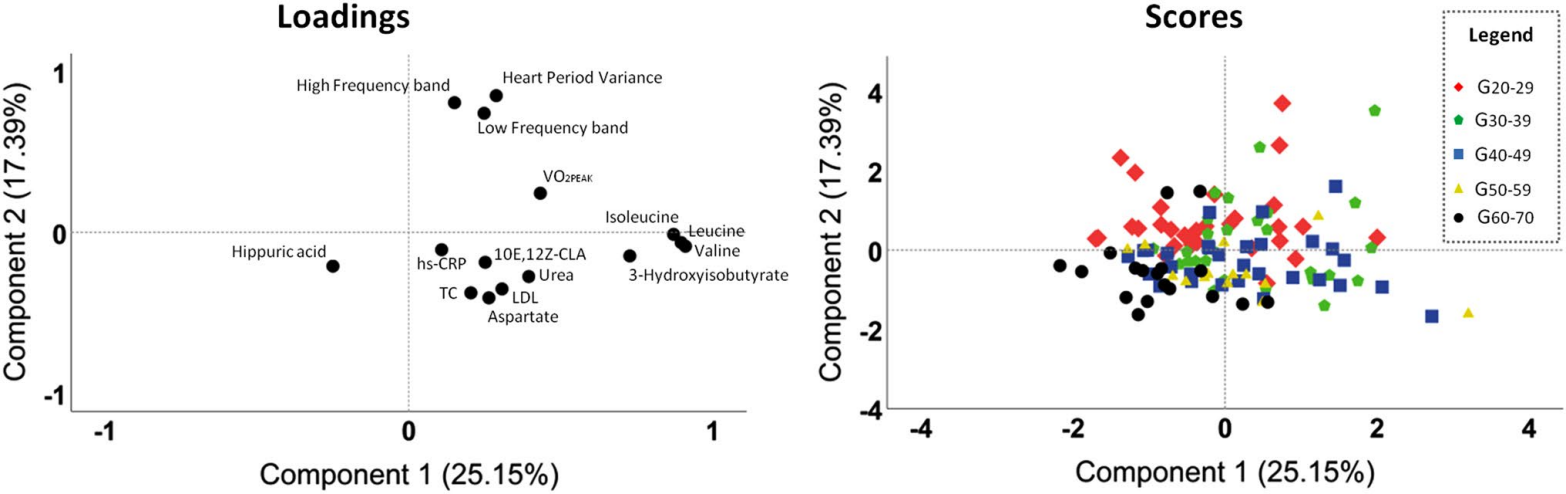
Principal component analysis in significant variables. *10E,12Z-CLA* 10E,12Z-octadecadienoic acid, *G*_*20–29*_ 20–29 years old group, *G*_*30–39*_ 30–39 years old group, *G*_*40–49*_ 40–49 years old group, *G*_*50–59*_ 50–59 years old group, *G*_*60–70*_ 60–70 years old group, *hs-CRP* high-sensitivity C-reactive protein, *LDL* low density lipoprotein, *TC* total cholesterol, *VO*_*2PEAK*_ peak oxygen consumption.

**Figure 3:**
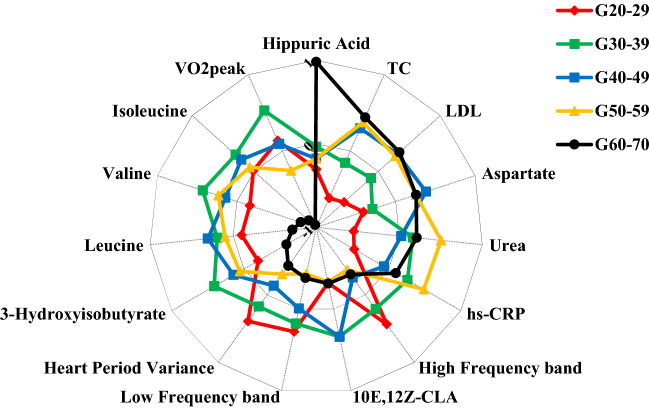
Characterization of each age group according to the significant variables. Data presented by z-score mean values of each age group for each variable. *10E,12Z-CLA* 10E,12Z-octadecadienoic acid, *G*_*20–29*_ 20–29 years old group, *G*_*30–39*_ 30–39 years old group, *G*_*40–49*_ 40–49 years old group, *G*_*50–59*_ 50–59 years old group, *G*_*60–70*_ 60–70 years old group, *hs-CRP* high-sensitivity C-reactive protein, *LDL* low density lipoprotein, *TC* total cholesterol, *VO*_*2PEAK*_ peak oxygen consumption.

The PCA analysis (Fig. 2, Supplementary Fig. S3) showed that hippuric acid was the most representative variable in the G_60–70_ as observed by the sample clustering in the lower left quadrant. The LF, HF, HP variance, and VO_2PEAK_ seem to be positively related (located in the upper right quadrant) and are reduced in the G_60–70_ due to the clustering of the samples being in the opposite quadrant. Moreover, branched-chain amino acids [BCAAs (leucine, isoleucine, and valine)] and 3-hydroxyisobutyrate seem to be positively related due to the proximity of these variables in the loading plot, and are strongly reduced in the G_60–70_ due to the clustering distance of the samples. Finally, aspartate, hs-CRP, urea, LDL, TC, and 10E,12Z-CLA seem to have a positive relationship as they are in the same quadrant, however, the relationship between aspartate, TC, and LDL is highlighted by the cluster of these variables in the loading plot. Similar results are observed in Fig. 3.

## Discussion

This was the first study that concurrently evaluated variables related to the metabolome, CAM, and CRF during the aging process in apparently healthy individuals. The results indicated that BCAAs (leucine, isoleucine, and valine), 3-hydroxyisobutyrate, hippuric acid, VO_2PEAK_, and global CAM are influenced by the healthy aging process and are mainly affected by changes that occur after the age of sixty. Serum levels of BCAAs and 3-hydroxyisobutyrate, and VO_2PEAK_ remained relatively stable until the G_50–59_ with a significant decrease in the last age group, while hippuric acid behaved contrary in the last age group (Table 2). Furthermore, CAM decreases with increasing age and is clearly reduced in the latter age group, with greater losses to CPM. Considering the integration of the results, we observed in PCA a greater representation of hippuric acid in the G_60–70_ and an indication of a lower representation of the other variables mentioned above for the same group compared to the other groups (Figs. 2, 3, Supplementary Fig. S3).

The amino acid metabolism in the aging process has been discussed for several years^40–42^. Some studies reported an increase in serum levels of most amino acids with the aging process, while others showed the opposite behavior^14,42^. The increase in serum amino acid levels during fasting is related to metabolic disorders and protein degradation^14,23,43^, and these conditions are often observed during aging as a result of changes in the nutritional and physical activity levels, increased oxidative stress and deoxyribonucleic acid (DNA) damage, and inflammation^2,44^. However, the serum levels of amino acid also are positively related to muscle mass level^44^ and a reduced amino acid level in serum during fasting may be related to low muscle mass^41,42^. BCAAs, in particular leucine, are important amino acids in regulating protein synthesis and degradation, as well as in muscle sensitivity to amino acid uptake, glycogen production, neurotransmitters, mitochondrial function, immune responses, telomere length, and oxidative stress control^40,44^. Most of these effects are due to the positive influence of BCAAs on the activation of the mammalian target of rapamycin (mTOR) protein^44^, whose effects of activation of this protein are still intensely discussed regarding the benefits and harms in the aging process^44^. However, a reduction in mTOR activity seems to be related to a longer life expectancy^16,45^.

One of the main findings of our study was the evidence of a reduction in serum BCAAs and 3-hydroxyisobutyrate levels in the oldest age group. This finding is in agreement with that observed in previous studies and may be related to a lower muscle mass in older individuals^41,42,46^. As all participants did not use continuous medication and did not have any disease, systemic changes, and risk factors (e.g., tobacco use, or excessive alcohol use, or obesity), the most advanced age groups probably consisted of individuals with good health, and therefore, serum reductions of BCAAs and 3-hydroxyisobutyrate are probably not related to pathological processes. Hippuric acid corroborates with this hypothesis as it was significantly higher in older individuals (Table 2) and because it is negatively related to insulin resistance and positively related to the production of anti-inflammatory and antiviral agents, and to the biodiversity and maturity of the gut microbiota^47,48^.

Blood and urinary hippuric acid levels tend to increase throughout healthy aging^47^ and it is related to renal function, as well as serum levels of creatinine and urea^49–51^. A serum increase of this metabolite could indicate impaired renal function^49,50^, however, recent studies have also proposed hippuric acid as a mark and a mediator of metabolic health^47^. This metabolite can be derived from the activity of the enriched gut microbiota on highly antioxidant and immunomodulatory compounds (such as polyphenols) found in fruit and vegetables and has protective effects on beta cells in the pancreas^47,48^. Gut microbiota enrichment, seen in individuals with high plasma/urinary hippuric acid levels, is also related to better nutrient absorption in the gut and has an inverse relationship with frailty syndrome in elderly people^47,52^. Therefore, it is likely that the increased serum hippuric acid levels in the G_60–70_ are more related to an enrichment of the gut microbiota than to renal function impairment as the urea and creatinine levels were within normal values in all age groups (Table 2, Supplementary Table S1)^39^.

Despite the increase in serum levels of hippuric acid in the last age group, which could indicate a favorable change, indices related to CAM (HF, LF, and HP variance) decrease with aging and presented the lowest values in the oldest age group, as seen in the study by Voss et al.^53^. This could be related to impairment in CAM in later age groups. Specifically, considering the spectral analysis, the reduction of both bands (LF and HF) implies a possible reduction of the CSM and especially a reduction in the CPM, since the LF represents a mixed component (CSM + CPM) with a sympathetic predominance, while the HF represents only the CPM^24,54^. The reduction in CPM that occurs with aging is a consequence of several factors such as structural and functional changes in cardiac myocytes, oxidative stress, inflammaging, impairment of regulatory mechanisms, and loss of interaction among subsystems^2,8,13,55,56^ which makes the cardiovascular system more susceptible to limitations, overloads, and cardiovascular diseases^13,53^.

In agreement with the impairments in CAM, we observed an increase in hs-CRP in older individuals^57^. Higher LDL and TC values were also observed in older age groups, but none of these indices had pathological values^38,58^. These findings are related to inflammaging and changes in lipid metabolism, as widely discussed in the literature in the context of aging^25,59^. In addition, we observed a possible positive relationship between cholesterol values (LDL and TC) and serum aspartate levels (Fig. 2), possibly due to the relationship of both to cardiovascular impairments^60,61^.

Aspartate is a non-essential amino acid and has a still uncertain relationship with the healthy aging process. It appears that there is an upward trend in serum non-essential amino acids^41^. Our results showed an increase in this amino acid in the G_40–49_ with a very slight reduction in the older age groups (ES_*d*_ < − 0.2 for both age groups: G_50–59_ and G_60–70_). This metabolite plays an important role in the production control of reactive oxygen species as it is essential in balancing oxidized nicotinamide adenine dinucleotide (NAD^+^)/reduced nicotinamide adenine dinucleotide (NADH) in mitochondria through the malate-aspartate shuttle^62^. It also participates in the urea cycle and can be characterized as an excitatory co-neurotransmitter along with glutamate at the *N*-Methyl-d-Aspartate receptor (NMDAR) in the central nervous system^40,63,64^. However, increases in serum aspartate have been observed in cardiovascular impairments^60^. Recent studies in rats have shown the presence of NMDAR in blood vessels and the heart, and its activation is related to a reduction in CPM and an increased susceptibility to cardiac arrhythmias^65^. However, unlike glutamate and homocysteine (which were not significant in our data)^66^, the role of aspartate on NMDAR in the cardiovascular system has not yet been verified.

It is expected that with advancing age there is a progressive decrease in the VO_2PEAK_^9^ due to several factors that involve functional, structural, and interactive changes in tissues and systems^5–7,56,67^. The VO_2PEAK_ was significantly lower in the G_60–70_ , indicating a lower ability to produce energy from oxygen. Among the several factors that contribute to the reduction in VO_2PEAK_ over aging^5–7,56,67^ the reduction in serum levels of BCAAs may be contributing to the reduction in oxygen consumption observed in the same group as BCAAs are related to protein synthesis and mitochondrial function^44,68,69^. Cardiovascular autonomic control may also have influenced the reduction in VO_2PEAK_ in the G_60–70_^67^, as we observed changes in CAM in this age group. With advancing age, there is a progressive reduction in cardiac output, especially due to the reduction in the maximum heart rate as a consequence of impaired beta-adrenergic stimulation of cardiac chronotropism^67^. However, it is noteworthy that CAM alterations were observed in the resting condition and peripheral factors for VO_2PEAK_ reduction (such as muscle mass reduction, mitochondrial dysfunction, and intracellular metabolic alterations) are also strongly influenced by aging^7,70^.

In summary, healthy aging provides metabolic, autonomic, and CRF changes. Reductions in muscle mass, compromised function and structure of organic systems, increased systemic inflammation and oxidative stress, accumulation of DNA damage, and imbalances in autonomic control are the main reasons discussed in the literature to explain these changes^2,5,8,13,71,72^. Moreover, the organism tries to counterbalance these changes with homeostatic mechanisms, which are limited and their depletion is related to the appearance of physiological limitations and diseases^2,3^. Thus, it is expected that with advancing age, the individual has a greater depletion of homeostatic mechanisms, and consequently, greater dependence on healthy habits for health maintenance^3^. In the present study, despite the marked reductions in CPM and CRF in the G_60–70_, a metabolic profile consistent with that observed in the healthy aging process discussed in the literature can be observed^42,47^. The reduction in BCAAs and 3-hydroxyisobutyrate, and the increase in hippuric acid are highlighted in the oldest age group and may be related to physiological processes and metabolic status that mitigate the deleterious effects existing in aging by lower activation of the mTOR protein^3,44^, by not contributing with the transport of fatty acids across the endothelium and insulin resistance^73^, and by the beneficial effects that high levels of hippuric acid may indicate^47,48^. However, considering the cross-sectional nature of the present study, it is impossible to conclude that the metabolic profile of individuals in the oldest group is a consequence of healthier habits throughout life.

This study is an observational and cross-sectional study and is based on a small sample when compared to other studies that address this topic^1,14,74^. However, this was the first study to assess the effects of healthy aging from an integrative perspective, considering metabolic and systemic activity markers (VO_2PEAK_ and autonomic control). In addition, two complementary techniques (NMR and LC–MS) were used to access the metabolome of each participant. The food record of the subjects included was not considered in the present study, and the subjects’ physical activity level was not measured. However, all subjects were under the same condition on the day of blood collection (12 h of fasting and with restriction of some foods/drinks, in addition to the restriction of performing strenuous physical activities), and were classified as apparently healthy according to strict criteria as described in the methodology section.

## Conclusion

The age group that showed the most significant alterations was the age group between 60 and 70 years old, where there was a reduction in serum levels of BCAAs and their derivative, as well as in CRF, in the global CAM (especially in CPM), and a significant increase in serum levels of hippuric acid. Thus, the metabolic profile, CRF, and CAM change as a result of aging impairments, however, some changes in the metabolic profile of apparently healthy older individuals without cardiovascular risk factors may be favorable to mitigating the deleterious effects of aging. Longitudinal studies are needed to assess the effects of good lifestyle habits on the metabolic profile, as well as on CRF and CAM, for a better understanding of healthy aging.

## Supplementary Information

Supplementary Information.

## Data Availability

The datasets generated during and/or analyzed during the current study are available from the corresponding author upon reasonable request.
