# Supplementary material for: Integrative perspective of the healthy aging process considering the metabolome, cardiac autonomic modulation and cardiorespiratory fitness evaluated in age groups
Source: Sci Rep. 2022 Dec 9;12:21314. doi: 10.1038/s41598-022-25747-5 (PMC9734749; doi:10.1038/s41598-022-25747-5)
Supplement: Supplementary file 1 — Supplementary Information. [file 41598_2022_25747_MOESM1_ESM.pdf]

Integrative perspective of the healthy aging process considering the metabolome, cardiac autonomic modulation and cardiorespiratory fitness evaluated in age groups

Étore De Favari Signini[1], Alex Castro[2], Patrícia Rehder-Santos[1], Juliana Cristina Millan-Mattos[1], Juliana Magalhães de Oliveira[2], Vinicius Minatel[1], Camila Bianca Falasco Pantoni[1,3]; Heloisa Sobreiro Selistre de Araújo[4]; Fernando Fabrizzi[5]; Alberto Porta[6,7]; Antônio Gilberto Ferreira[2]; Regina Vincenzi Oliveira[2]; Aparecida Maria Catai[1,*].

[1] Department of Physiotherapy, Federal University of São Carlos, São Carlos, São Paulo, Brazil.

[2] Department of Chemistry, Federal University of São Carlos, São Carlos, São Paulo, Brazil.

[3] Department of Gerontology, Federal University of São Carlos, São Carlos, São Paulo, Brazil

[4] Departament of Physiological Sciences, Federal University of São Carlos, São Carlos, São Paulo, Brazil.

[5] Penápolis Educational Foundation (FUNEPE), Penápolis, São Paulo, Brazil.

[6] Department of Biomedical Sciences for Health, University of Milan, Milan, Italy.

[7] Department of Cardiothoracic, Vascular Anesthesia and Intensive Care, Policlinico San Donato, San Donato Milanese, Milan, Italy.

Keywords: Metabolomics, Autonomic Nervous System, Oxygen Consumption, Healthy Aging.

Corresponding author: Aparecida Maria Catai, PhD, Department of Physical Therapy, Cardiovascular Physical Therapy Laboratory, Nucleus of Research in Physical Exercise, Federal University of São Carlos, Via Washington Luiz, km 235, CP: 676, 13565-905, São Carlos-SP, Brazil, Tel: +55 16 33518705, Fax: +55 16 33612081, email: mcatai@ufscar.br.
Co-corresponding author: signinietof@gmail.com (Étore De F. Signini)

Competing Interests: The authors have no competing interest to declare.

SUPPORTING INFORMATION

Supplementary Table S1. Biochemical blood tests.

Supplementary Table S2. Cardiac autonomic control during the aging process.

Supplementary Table S3. Compounds identified on ^1H NMR and submitted to statistical analysis considering the age groups.

Supplementary Table S4. Chemical structure and chemical shift (δ - ppm) of metabolites that showed statistical significance during the aging process observed in ^1H NMR.

Supplementary Table S5. Features detected on LC-HRMS and submitted to statistical analysis considering the age groups.

Supplementary Table S6. LC-HRMS data of the statistically significant features identified in serum samples.

Supplementary Figure S1. Instrumental stability assessed through quality control samples.

Supplementary Figure S2: Representation of the serum 1D ^1H NMR spectrum region where the main metabolites were quantified for a single participant.

Supplementary Figure S3. Principal components analysis considering the five main components with eigenvalues greater than 1 for all significant variables between the age groups.

Supplementary Table S1. Biochemical blood tests.

**Characteristics**	**All**	**G_{20-29}**	**G_{30-39}**	**G_{40-49}**	**G_{50-59}**	**G_{60-70}**	***P-value***
TC (mg/dL)	186.50 (162.00 — 203.00)	171.00 (139.00 — 189.00)	179.00 (159.00 — 197.50)	191.50 (177 — 213.25)	203.00 (170.25 — 207.00)	194.00 (171.00 — 213.00)	**<0.001**
HDL (mg/dL)	54.00 (44.00 — 66.00)	50 (44.00 — 67.00)	53.00 (44.00 — 62.50)	50.00 (42.50 — 66.00)	61.00 (43.50 — 64.50)	59.50 (54.00 — 65.50)	0.688
LDL (mg/dL)	109.50 (90.25 — 123.00)	93.00 (65.00 — 113.00)	102.00 (91.00 — 121.50)	118.50 (94.00 — 130.25)	115.00 (110.00 — 132.50)	110.00 (100.50 — 131.50)	**0.003**
VLDL (mg/dL)	18.00 (14.00 — 25.00)	15.00 (12.00 — 21.00)	18.00 (14.00 — 24.00)	20.00 (14.00 — 27.00)	21.00 (16.00 — 26.50)	19.00 (14.25 — 23.75)	0.413
Triglycerides (mg/dL)	90.50 (68.00 — 126.75)	75.00 (60.00 — 105.00)	89.00 (68.50 — 120.50)	99.00 (68.25 — 136.50)	104.00 (81.00 — 131.25)	96.00 (69.25 — 118.50)	0.437
Uric acid (mg/dL)	5.10 (4.30 — 6.10)	4.80 (4.40 — 5.80)	5.00 (4.23 — 6.00)	5.55 (4.65 — 6.40)	5.15 (4.20 — 7.23)	4.70 (4.23 — 5.55)	0.252
Creatinine (mg/dL)	0.89 (0.76 — 1.00)	0.84 (0.72 — 0.98)	0.95 (0.76 — 1.06)	0.95 (0.79 — 1.03)	0.89 (0.80 — 0.94)	0.83 (0.76 — 0.89)	0.342
Fasting glucose (mg/dL)	91.00 (86.00 — 95.00)	87.00 (83.00 — 93.00)	88.00 (85.00 — 92.00)	92.00 (89.00 — 96.50)	93.50 (90.00 — 95.00)	93.50 (87.50 — 96.75)	0.014
Urea (mg/dL)	31.50 (27.00 — 36.00)	27.00 (23.00 — 32.00)	33.00 (29.50 — 37.00)	32.00 (29.00 — 36.50)	34.00 (30.75 — 37.75)	32.50 (29.25 — 36.50)	**0.006**
hs-CRP (mg/L)	0.48 (0.16 — 1.18)	0.21 (0.07 — 0.50)	0.76 (0.24 — 1.51)	0.37 (0.16 — 0.97)	1.19 (0.50 — 1.81)	0.66 (0.23 — 1.13)	**0.003**

Data presented in median and interquartile interval. The logarithm (for VLDL and Triglycerides), quadratic (for TC), root square (for creatinine), and root cubic (for HDL and hs-CRP) transformations were used in the statistics. The variables not listed above were used the original data. G_{20-29}: 20-29 years old group; G_{30-39}: 30-39 years old group; G_{40-49}: 40-49 years old group; G_{50-59}: 50-59 years old group; G_{60-70}: 60-70 years old group; HDL: high density lipoprotein; hs-CRP: high-sensitivity C-reactive protein; LDL: low density lipoprotein; TC: Total cholesterol; VLDL: very low density lipoprotein. One-way ANOVA with *P* <0.01.

Supplementary Table S2. Cardiac autonomic control among different age groups.

Variables	G_{20-29}	G_{30-29}	G_{40-49}	G_{50-59}	G_{60-70}	***P-value***
Time domain Analysis						
HP mean $_{(ms)}$	911.86 (828.53 – 983.22)	942.06 (854.99 – 1063.28)	924.40 (849.07 – 1026.68)	933.27 (891.21 – 1028.35)	975.17 (852.88 – 1056.47)	0.430
HP Variance $_{(ms^2)}$	2210.29 (1560.85 – 3355.20)	1709.86 (929.65 – 3496.37)	1345.62 (915.38 – 2210.50)	1189.98 (844.33 – 1843.30)	825.30 (557.82 – 1243.27)	**0.002**
Spectral Analysis						
$LF_{(ms^2)}$	811.26 (476.83 – 1302.74)	677.18 (264.04 – 1075.83)	460.33 (271.12 – 983.84)	373.88 (204.14 – 445.24)	176.44 (72.29 – 385.29)	**<0.001**
$HF_{(ms^2)}$	798.68 (429.77 – 1854.86)	517.02 (326.89 – 970.09)	340.34 (188.32 – 589.96)	364.90 (122.54 – 627.22)	176.38 (93.68 – 359.37)	**0.001**
$LF_{(nu)}$	45.37 (35.49 – 59.70)	49.39 (35.78 – 65.73)	60.43 (45.47 – 73.48)	56.53 (41.85 – 75.13)	44.91 (34.74 – 59.67)	0.460
$HF_{(nu)}$	51.67 (37.57 – 64.22)	50.46 (33.88 – 63.11)	39.48 (26.07 – 53.48)	42.88 (24.37 – 57.76)	53.52 (39.79 – 63.66)	0.524
$LF/HF_{(ratio)}$	0.88 (0.55 – 1.59)	0.98 (0.56 – 1.96)	1.55 (0.85 – 2.82)	1.32 (0.73 – 3.15)	0.84 (0.55 – 1.51)	0.635

Data presented in median and interquartile interval. The logarithm (HP mean, HP Variance, $LF_{ms}{}^2$, $HF_{ms}{}^2$, and LF/HF) transformation was used in the statistics. The variables not listed above were used the original data. G_{20-29}: 20-29 years old group; G_{30-39}: 30-39 years old group; G_{40-49}: 40-49 years old group; G_{50-59}: 50-59 years old group; G_{60-70}: 60-70 years old group; HF: high frequency band; HP: heart period; LF: low frequency band. One-way ANOVA with P <0.01.

Supplementary Table S3. Compounds identified on ^{1}H NMR and submitted to statistical analysis considering the age groups.

**Metabolite**	**Data transformation**	***P-value***
Valine	Inverse	**<0.001**
Isoleucine	Natural Logarithm	**<0.001**
3-Hydroxyisobutyrate	Natural Logarithm	**0.003**
Leucine	Natural Logarithm	**0.003**
Aspartate	Original	**0.007**
Choline	Original	0.018
Dimethyl sulfone	Square Root	0.025
Lactate	Natural Logarithm	0.030
2-Hydroxyisovalerate	Natural Logarithm	0.041
Serine	Quadratic	0.043
Succinate	Natural Logarithm	0.044
Threonine	Cubic Root	0.046
Acetoacetate	Natural Logarithm	0.062
Ornithine	Original	0.067
Phenylalanine	Natural Logarithm	0.079
Glutamine	Original	0.082
Creatinine	Original	0.093
Methionine	Natural Logarithm	0.131
Betaine	Cubic Root	0.160
Formate	Inverse	0.171
Lysine	Square Root	0.179
2-Hydroxybutyrate	Natural Logarithm	0.232
N,N-Dimethylglycine	Square Root	0.242
Glucose	Natural Logarithm	0.294
Alanine	Original	0.308
Anserine	Natural Logarithm	0.330
3-Hydroxybutyrate	Natural Logarithm	0.389
2-Oxoisocaproate	Original	0.410
Tyrosine	Inverse	0.437
Pyruvate	Cubic Root	0.461
Asparagine	Original	0.495
Dimethylamine	Original	0.520
Ascorbate	Square Root	0.555
Glycine	Original	0.572
Proline	Natural Logarithm	0.585
Histidine	Quadratic	0.732
O-Acetylcarnitine	Original (non-parametric)	0.759*
2-Oxoglutarate	Original	0.790
3-Methyl-2-oxovalerate	Original	0.794
Trimethylamine	Square Root	0.809
Glycerol	Natural Logarithm	0.812
Acetate	Natural Logarithm	0.829
Citrate	Square Root	0.899
Hypoxanthine	Natural Logarithm	0.921
Sarcosine	Natural Logarithm	0.924
Creatine	Cubic Root	0.932
Ethanol	Inverse	0.989

*Kruskal-Wallis test. One-way ANOVA with $P < 0.01$.

Supplementary Table S4. Chemical structure and chemical shift (δ - ppm) of metabolites that showed statistical significance during the aging process observed in ^{1}H NMR.

Nº	Compounds and Structure	δ ^{1}H ppm (Multiplicity*, Nº H, J)	COSY $^{1}H/^{1}H$	HSQC $^{13}C/^{1}H$	HMBC
**1**	Aspartate 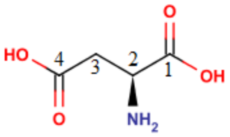	**1.** ---<br>**2.** 3.88-3.90 (m – 1H)<br>**3.** 2.65 (dd – 2H, 17.5 Hz, 8.88 Hz)<br>**3.** 2.80 (dd – 2H, 17.5 Hz, 3.64 Hz)<br>**4.** ---	**2.** 3.88-3.90/2.65<br>**2.** 3.88-3.90/2.80	Unobserved	**3.** 182.0
**2**	Isoleucine 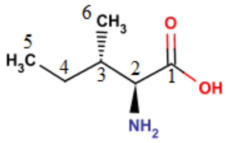	**1.** ---<br>**2.** 3.66 (d – 1H)<br>**3.** 1.94-2.00 (m – 1H)<br>**4.** 1.24-1.45 (m – 2H)<br>**5.** 0.92 (t – 3H)<br>**6.** 0.99 (d – 3H, 7.02 Hz)	**3.** 1.94-2.00/1.45<br>**5.** 0.92/1.45<br>**6.** 0.99/1.94-2.00	**2.** 62.7/3.66<br>**5.** 13.8/0.92<br>**6.** 17.6/0.99	**5.** 27.7<br>**6.** 27.7; 38.9; 62.7
**3**	Leucine 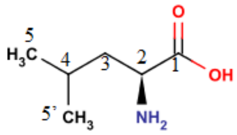	**1.** ---<br>**2.** 3.71-3.73 (m – 1H)<br>**3.** 1.65-1.75 (m –2H)<br>**4.** 1.65-1.75 (m – 1H)<br>**5.** 0.94 (d – 3H, 6.60 Hz)<br>**5'.** 0.95 (d – 3H, 6.60 Hz)	**2.** 3.71-3.73/1.72<br>**5.** 0.94/1.69<br>**5'.** 0.95/1.69	**2.** 56.2/3.71-3.73<br>**3.** 27.0/1.65-1.75<br>**4.** 42.8/1.65-1.75<br>**5.** 24.7/0.94<br>**5'.** 24.7/0.95	**3.** 42.8<br>**5.** 24.7; 27.0; 42.8<br>**5'.** 24.7; 27.0; 42.8
**4**	3-Hydroxyisobutyrate 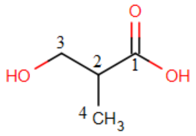	**1.** ---<br>**2.** 2.44-2.50 (m – 1H)<br>**3.** 3.51-3.55 (m – 2H)<br>**4.** 1.06 (d – 3H, 7.15 Hz)	**2.** 2.44-2.50/1.06	**3.** 3.51-3.55/65.5<br>**4.** 1.06/19.1	**3.** 175.4
**5**	Valine 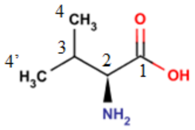	**1.** ---<br>**2.** 3.59 (d – 1H, 4.50 Hz)<br>**3.** 2.22-2.30 (m)<br>**4.** 0.97 (d – 3H, 7.00 Hz)<br>**4'.** 1.03 (d – 3H, 7.00 Hz)	**4.** 0.97/2.26<br>**4'.** 1.03/2.26	**2.** 63.3/3.59<br>**3.** 31.3/2.22-2.30<br>**4.** 19.7/0.97<br>**4'** 20.9/1.03	**4.** 20.9; 31.3; 63.3<br>**4'.** 19.7; 31.3; 63.3

*Inferred multiplicity based on the Chenomx library. Singlet (s); doublet (d); triplet (t); multiplet (m).

Supplementary Table S5. Features detected on LC-HRMS and submitted to statistical analysis considering the age groups.

m/z	**Ionization mode**	**Data transformation**	***P-value***
178.0508	Negative	Natural Logarithm	**<0.001**
129.0560	Negative	Inverse	**<0.001**
298.2743	Positive	Natural Logarithm	**0.001**
537.4164	Negative	Natural Logarithm	**0.004**
872.6302	Positive	Inverse	**0.009**
593.4791	Negative	Cubic Root	**0.009**
586.3139	Negative	Natural Logarithm	0.011
655.2784	Positive	Natural Logarithm	0.012
467.3731	Negative	Natural Logarithm	0.015
696.5247	Positive	Natural Logarithm	0.019
144.9234	Negative	Original (non-parametric)	0.021*
145.0619	Negative	Natural Logarithm	0.021
916.6549	Positive	Natural Logarithm	0.025
100.9334	Negative	Square Root	0.028
868.5977	Positive	Inverse	0.043
652.4982	Positive	Inverse	0.044
167.0212	Negative	Square Root	0.052
293.1759	Negative	Cubic Root	0.061
698.7851	Positive	Cubic Root	0.065
1086.1771	Positive	Natural Logarithm	0.066
617.7374	Negative	Original	0.068
731.3168	Negative	Square Root	0.075
195.0873	Positive	Cubic Root	0.078
203.0824	Negative	Cubic Root	0.078
191.0196	Negative	Original	0.091
619.7504	Positive	Original	0.092
195.8108	Negative	Original (non-parametric)	0.092*
599.3807	Positive	Inverse	0.103
942.3430	Negative	Natural Logarithm	0.109
173.0816	Negative	Natural Logarithm	0.122
564.4467	Positive	Inverse	0.130
612.3302	Negative	Natural Logarithm	0.138
853.5210	Positive	Quadratic	0.138
415.2107	Positive	Cubic Root	0.156
117.0555	Negative	Natural Logarithm	0.158
284.2944	Positive	Natural Logarithm	0.160
256.2635	Positive	Original (non-parametric)	0.178*
384.3453	Positive	Quadratic	0.188
187.0973	Negative	Natural Logarithm	0.200
484.2277	Negative	Square Root	0.241
199.8048	Negative	Original (non-parametric)	0.279*
853.5209	Positive	Quadratic	0.282
663.2650	Positive	Original	0.299
686.4438	Positive	Inverse	0.300
130.0872	Negative	Original	0.302
359.3168	Positive	Cubic Root	0.313
380.2553	Positive	Natural Logarithm	0.330
381.1734	Negative	Original	0.331
187.0071	Negative	Cubic Root	0.344
281.2480	Negative	Original	0.348
228.1953	Positive	Natural Logarithm	0.371
702.5082	Positive	Cubic Root	0.381
766.4569	Positive	Quadratic	0.410
162.8390	Negative	Original	0.412
648.3784	Positive	Natural Logarithm	0.427
603.7898	Positive	Square Root	0.455
696.7722	Negative	Original	0.457

Supplementary Table S5. Cont.

m/z	Ionization Mode	Data transformation	*P-value*
350.2103	Positive	Natural Logarithm	0.458
746.4421	Positive	Original	0.459
310.3093	Positive	Inverse	0.467
824.4991	Negative	Original	0.482
432.2786	Positive	Square Root	0.491
737.4358	Positive	Original	0.498
686.3570	Positive	Natural Logarithm	0.515
628.4038	Positive	Original (non-parametric)	0.527*
604.3523	Positive	Natural Logarithm	0.539
89.0242	Negative	Original	0.544
744.4863	Positive	Inverse	0.554
708.4155	Positive	Original	0.557
128.0353	Negative	Square Root	0.563
540.3310	Negative	Quadratic	0.572
282.2792	Positive	Original	0.574
112.9856	Negative	Inverse	0.595
180.0665	Negative	Natural Logarithm	0.609
355.1578	Negative	Original	0.614
164.0714	Negative	Inverse	0.623
183.0840	Positive	Cubic Root	0.625
145.0504	Negative	Original (non-parametric)	0.626*
388.2532	Positive	Natural Logarithm	0.626
766.4569	Positive	Quadratic	0.635
426.3569	Positive	Natural Logarithm	0.641
568.3611	Negative	Natural Logarithm	0.667
255.2325	Negative	Natural Logarithm	0.667
833.5036	Positive	Original	0.668
383.1894	Negative	Original (non-parametric)	0.670*
283.2637	Negative	Quadratic	0.676
452.2026	Positive	Cubic Root	0.679
824.4991	Positive	Quadratic	0.685
773.5058	Positive	Inverse	0.723
426.1868	Positive	Natural Logarithm	0.728
164.8360	Negative	Square Root	0.732
802.5591	Negative	Inverse	0.733
395.2744	Negative	Original	0.736
337.1666	Positive	Natural Logarithm	0.738
1102.1848	Positive	Original	0.745
653.2665	Negative	Original	0.748
614.3443	Negative	Natural Logarithm	0.763
452.2029	Positive	Natural Logarithm	0.763
378.2411	Negative	Inverse	0.769
118.0863	Positive	Original	0.790
795.4797	Positive	Original	0.791
158.9781	Negative	Original	0.810
303.2325	Negative	Natural Logarithm	0.815
119.0863	Positive	Square Root	0.817
158.1537	Positive	Cubic Root	0.823
516.3006	Positive	Natural Logarithm	0.828
717.7568	Positive	Original	0.830
532.2993	Negative	Original	0.853
775.4643	Positive	Original	0.855
795.4788	Positive	Original (non-parametric)	0.862*
103.0398	Negative	Square Root	0.868
147.0759	Positive	Original	0.873
538.3141	Positive	Natural Logarithm	0.879
279.2327	Negative	Cubic Root	0.900
657.4222	Positive	Cubic Root	0.904

Supplementary Table S5. Cont.

m/z	Ionization Mode	Transformation used	*P-value*
583.2555	Negative	Natural Logarithm	0.910
583.2560	Negative	Natural Logarithm	0.912
379.1576	Negative	Square Root	0.916
476.2775	Negative	Natural Logarithm	0.923
271.2270	Negative	Natural Logarithm	0.943
161.0955	Positive	Natural Logarithm	0.955
560.3259	Positive	Cubic Root	0.958
636.2596	Negative	Original	0.992
692.4049	Positive	Original (non-parametric)	0.994*
500.2775	Negative	Natural Logarithm	0.996

*Kruskal-Wallis test. One-way ANOVA with $P < 0.01$.

Supplementary Table S6. LC-HRMS data of the statistically significant features identified in serum samples.

**Features**	**HMDB code**	**Formula**	**Monoisotopic Mass**	**Adduct**	**Adduct *m/z***	**Error (ppm)**
Hippuric Acid	HMDB0000714	$C_9H_9NO_3$	179.0582	$[M-H]^-$	178.0508	1
10E,12Z-CLA	HMDB0005048	$C_{18}H_{32}O_2$	280.2402	$[M+NH_4]^+$	298.2743	1
Unknown	-	-	-	Negative	129.0560	-
Unknown	-	-	-	Negative	537.4164	-
Unknown	-	-	-	Positive	872.6302	-
Unknown	-	-	-	Negative	593.4791	-

10E,12Z-CLA: 10E,12Z-octadecadienoic acid.

Supplementary Figure S1. Instrumental stability assessed through quality control samples.

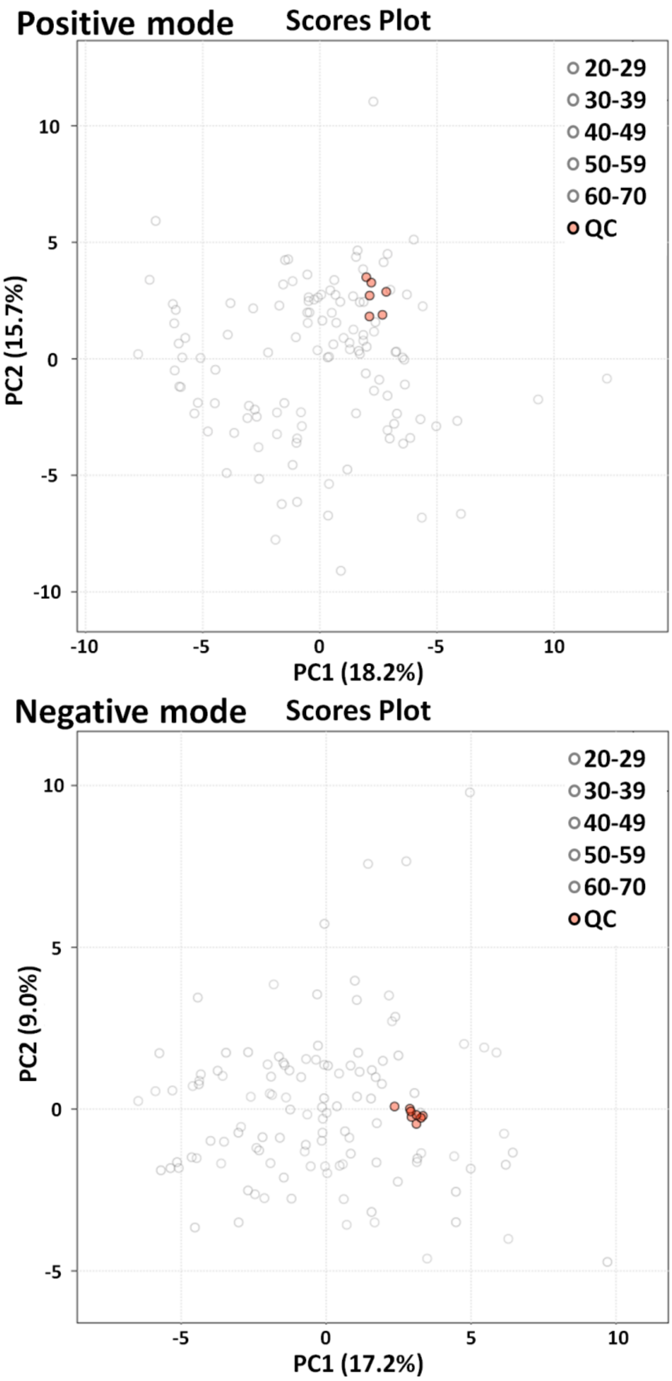

The missing data were replaced by the feature mean and the data were submitted to auto scaling (mean-centered and divided by the standard deviation of each variable). All other treatments were previously performed in Excel software (exclusion of features with missing data > 10%, with values similar to blank samples, and with a coefficient of variation in QCs greater than 20%). QC: quality control samples; PC: principal component. Data processed in *Metaboanalyst 5.0* software (https://www.metaboanalyst.ca/).

Supplementary Figure S2: Representation of the serum 1D ^{1}H NMR spectrum region where the main metabolites were quantified for a single participant.

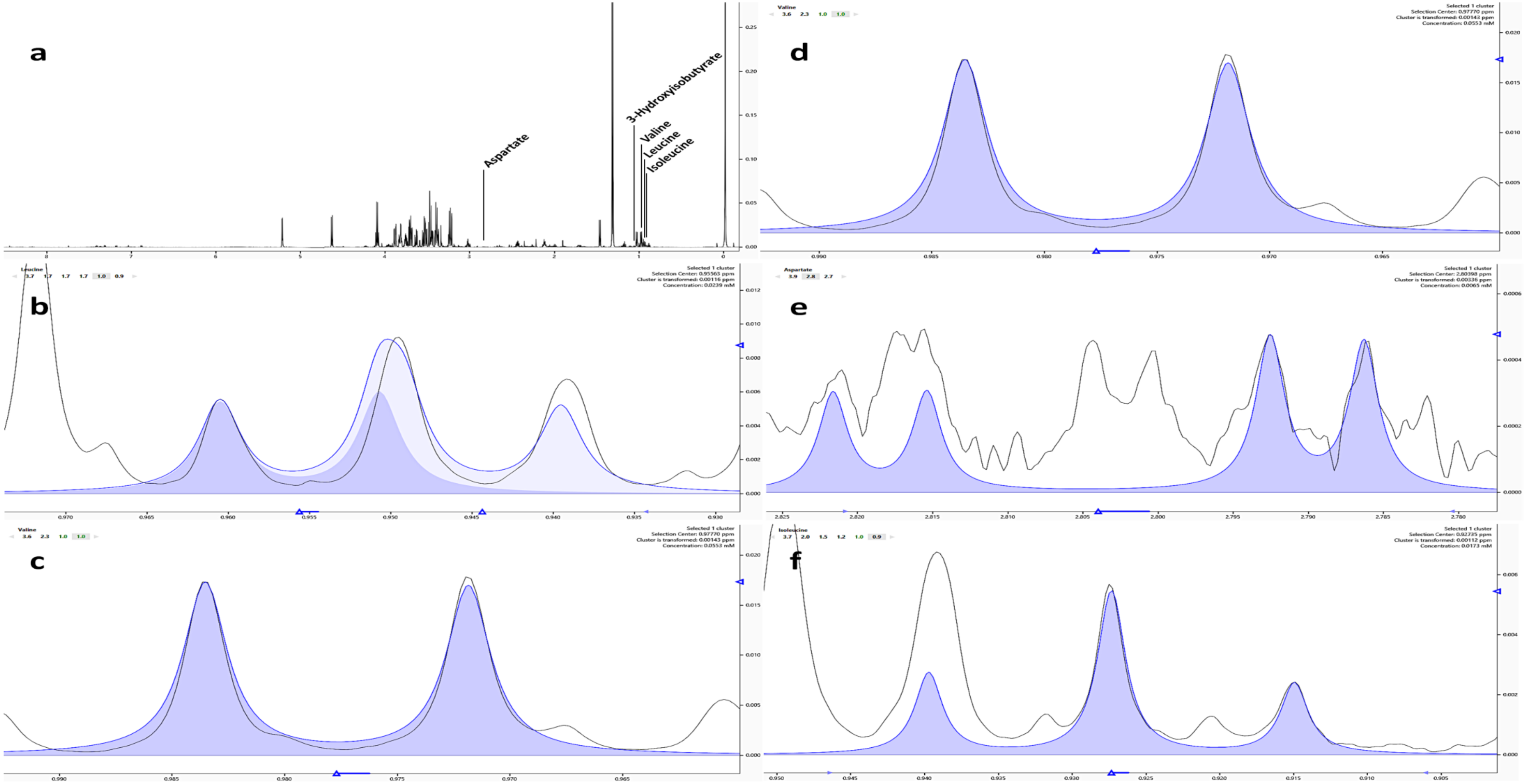

(**a**): Entire Spectrum; (**b**): Leucine; (**c**): Valine; (**d**): 3-Hydroxyisobutyrate; (**e**): Aspartate; (**f**): Isoleucine.

Supplementary Figure S3. Principal components analysis considering the five main components with eigenvalues greater than 1 for all significant variables between the age groups.

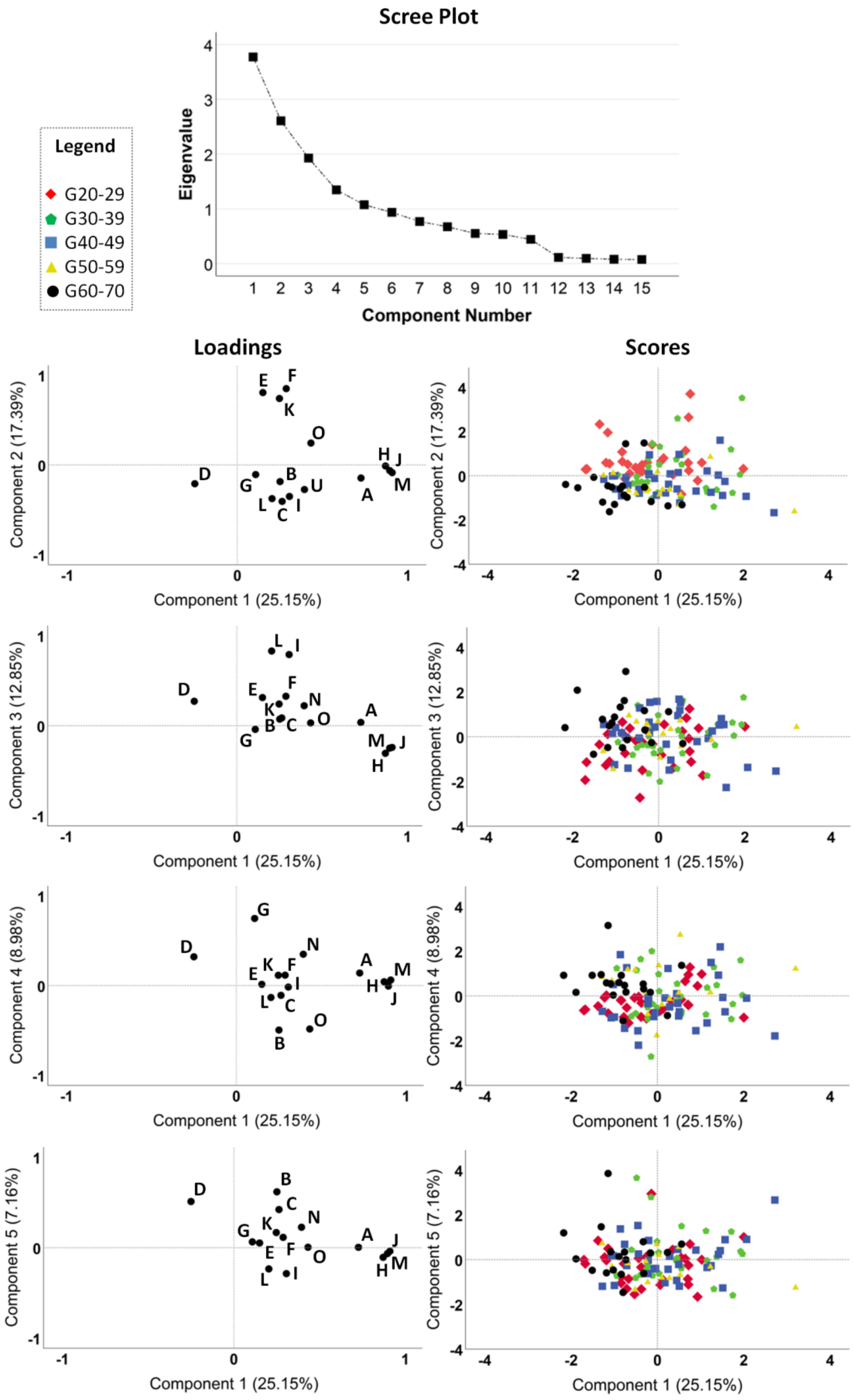

A: 3-Hydroxyisobutyrate; B: 10E,12Z-octadecadienoic acid; C: Aspartate; D: Hippuric acid; E: high frequency band; F: heart period variance; G: high-sensitivity C-reactive protein; H: Isoleucine; I: low density lipoprotein; J: Leucine; K: low frequency band; L: Total cholesterol; M: Valine; N: urea; O: peak oxygen consumption.
